# Clinical Studies of Some Suppurative Diseases of the Maxillæ

**Published:** 1899-03

**Authors:** Henry C. Boenning

**Affiliations:** Philadelphia


### CLINICAL STUDIES OF SOME SUPPURATIVE DISEASES OF THE MAXILLÆ.[1]

BY HENRY C. BOENNING, M.D., PHILADELPHIA.[2]

From the point of construction the maxillæ are the most important bones of the face, inasmuch as they form a large part of the osseous framework of this part of the skeleton; but their greatest importance is due, of course, to the fact that they contain the teeth, and to their relations to important organs such as the

---

[1] Read before the Academy of Stomatology, December 27, 1898.

[2] Professor of Anatomy and Surgery in the Philadelphia Dental College; Quarantine Physician of Pennsylvania; Surgeon to the Garretson Hospital, etc., etc.

eyes, nose, and mouth. A number of years ago, when my attention was first directed to the diseases of the maxillary bones associated with suppuration, notably alveolar abscess and antral empyema, I observed that while a great number of these cases were being constantly treated at the dispensary, very few of them progressed towards a final successful issue, and still fewer were cured. The suppurative troubles were mostly recurrent, and, though the diseases may have been modified and apparently cured, in a very little while the cases would again appear with a return of the original trouble.

It may be proper for me to say that I owe a great debt of gratitude to the Philadelphia School of Anatomy, which I conducted from 1883 to 1896, inclusive, inasmuch as frequent illustrations of the suppurative diseases of the maxillæ presented themselves on the dissecting-table, and I never lost an opportunity to examine every cadaver for specimens in morbid anatomy illustrating alveolar abscess, antral empyema, and other pathological conditions of the maxillæ. I was wonderfully rewarded in my studies on these diseases by finding (especially during the years from 1887 to 1894, while I was most active in this direction) a large number of illustrations in morbid anatomy of alveolar abscess, and quite a series of specimens of antral disease, which I took especial care to study very carefully.

In many of the specimens I secured, and which I still have in my possession, I found that the teeth had received very careful dental treatment, showing in many instances filling-material extending well down the pulp-canals. In almost every case of section upon suspected cases of alveolar abscess, and, in many instances, sections of the alveolar structures where no suspicion of alveolar abscess existed, I was rewarded by finding well-formed pus-cavities, in some of which the morbid appearances were not only interesting, but instructive to the highest degree.

In specimens of alveolar abscess where the lesions were marked there were present, at and near the extremity of the roots of the affected teeth, cavities of greater or less dimensions, filled in some instances with putrid pus, or pus undergoing caseous transformation, with flakes of carious bone, shreds of tissue, inspissated *débris*, and in several instances the apices of the roots were found roughened, eroded,—in a word, necrotic. In these appearances, I concluded, lay the reason why, even with careful treatment, alveolar abscess continues indefinitely as a chronic and recurrent disease.

The pathological accumulations are retained within the abscess cavity in the substance of the bone, and although (as was shown by the appearance of the teeth in the sections) careful enlargement of the canals of the roots with the conventional treatment of the disease is practised, the contents of the abscess cavities are of such character, substance, and size that it is physically impossible to force them out of the small openings through the roots. When it is considered that such treatment involves, sometimes, the destruction of the normal nerve-supply of the tooth, the enlargement, perhaps, of the neural canal and of its apical opening, and that it is sought through the channel thus formed to discharge the contents of the alveolo-dental abscess, then, I say, the reason why such diseases are recurrent and seldom cured by this line of treatment is clearly apparent. How is it possible to discharge the contents of an alveolo-dental abscess through the minute canal ordinarily cleared for the purpose of tapping alveolar abscess, when the products that are to be discharged are, as a rule, many times larger than the canal which is formed for the purpose of the evacuation of the morbid material?

Following these examinations, or, rather, associated with them, I followed with great care the clinical service in our dental infirmary, and found, as a rule, that alveolar abscess was regarded as one of the most intractable of complaints, recurrent and unsatisfactory in many cases. I inquired very carefully at the dispensaries of other large dental schools, and the testimony was the same.

When nature opens an abscess in any part of the body, of course spontaneously, it results in a prompt cure *if the opening is sufficiently large.* It occurred to me that it has been the practice of surgery, when desiring to evacuate an acute abscess, and, in fact, any accumulation of pus (except such as occurs in the development of a cold abscess), to make a free, bold incision right down to the purulent accumulation, and thus at once discharge not only the pus, but the colonies of developing micro-organisms, the infected material and *débris,* any necrotic masses, and everything incidental to the development of a disease of this kind.

I considered, furthermore, that it was the practice of surgery, in the treatment of abscesses associated with the osseous structures, to cut down upon the seat of pus-formation within the bone, and then by trephine, or otherwise, make an opening sufficiently large to insure prompt, free, and satisfactory discharge of the contents of such a cavity. I asked myself, why can we not apply the same rule of surgery to the treatment of alveolo-dental abscess? Why shall we temporize with any given case of alveolo-dental abscess, associated probably with a necrotic root, by attempting the discharge of the pus and other contents of the abscess cavity by a minute canal drilled through the root of the tooth, with little chance or hope of success, and often with the probability, if not assurance, of recurrence? Although alveolar abscess in the great majority of cases is preceded by the death of the pulp, such is not invariably the case, but in these exceptional cases the pulp is by the conventional treatment destroyed, of course.

Following my conviction in this matter, it became my plan to urge a radical treatment of alveolo-dental abscess. Instead of enlarging the pulp-canals and treating the abscess through them, I practised opening into the abscess through the external alveolar plate. This operation is so very easily performed that it is readily practised by every dental surgeon. I have found that the most satisfactory plan to follow is, first, to anæsthetize the part or patient; next, to antisepticize the parts and then raise a periosteal flap from the external alveolar plate; the bone being exposed, put a clean bur of sufficient size in the holder of the surgical engine and drill directly into the alveolar abscess, then completely remove its contents; all of the accumulated *débris* and, if such be the case, the necrotic extremity of the root should be removed. Not that I would ask you to forego treatment through the root-canals whenever alveolar abscess occurs; not at all. In fact, be sure and put the affected tooth in the best possible condition after opening and thoroughly discharging the abscess through the external alveolar plate, but do this first. In alveolo-dental abscess there is not simply a pus formation at the apex of a root and infection of the contiguous structures, but there is at the same time infiltration of pyogenic micro-organisms and their products throughout the entire soft structures in the proximity of the abscess cavity.

Basing my opinion upon experience, I think that the most satisfactory treatment of this affection is an external opening into the abscess cavity by means of a bur from one-eighth to one-quarter of an inch in diameter; next, thorough antisepsis of the abscess cavity; then the proper sterilization of the pulp-canals and the filling of the same; and then packing the abscess cavity with iodoform gauze or other dressing, leaving the opening through the external alveolar plate lightly covered by the pendent fold formed by the periosteal flap. This becomes attached, and very soon the wound closes.

What manner of objection can be urged against such surgery as this? None whatever; it is a line of treatment which nature not infrequently adopts. How often do we see infected ostitis and periostitis followed by softening of the bone and caries, even necroses, the development of large abscesses in the overlying soft structures, and unsightly sinuses presenting at some part of the face external to, and sometimes at a very considerable distance from, the seat of the disease? It is the line of treatment which is entirely in harmony with that practised by every advanced surgeon for the treatment of abscess in other parts of the human body. There is but a single exception to the treatment of any abscess by a full, free, large opening, and that is *cold abscess* associated, let us say, with deep-seated structures, as with the bodies of the vertebræ. Here, of course, we can understand why great care and measures involving the smallest kind of an opening are imperatively required. Tubercular or cold abscess, as, for instance, psoas abscess, is due primarily to the action of the bacillus tuberculosis, and only to a certain degree to certain pyogenic micro-organisms. It is different in its nature and its course from an acute abscess or a chronic abscess, or any pus formation which can be compared to an ordinary alveolar abscess. To open a psoas abscess, or a cold abscess, where the greater part of the sac is concealed and beyond our reach, by a large, free opening, might simply allow the entry of germs or putrefaction and decomposition, and result in the formation of such toxines as would destroy the individual. Surgical annals afford many illustrations of the truth of this statement.

Such an abscess, however, may occur in the alveolar structures, but it is then limited and may require the same treatment as has been laid down for ordinary alveolar abscess. In fact, cold abscess in the alveolar regions can occur, but, as has been said, the treatment, owing to its accessibility and superficial character, requires a free opening and thorough evacuation and curettement.

The treatment ordinarily followed for the cure of antral empyema has generally been the removal of a molar, diseased or otherwise, and then through some convenient socket perforation of the floor and irrigation of the antrum. Who that has had large experience does not remember numerous cases of chronic antral disease that have been recurrent through a period of many years? The case-book at my clinic will give the accurate details of a number of cases of antral empyema which have existed anywhere from two to twenty years, during all that time having been under the treatment of numerous dental practitioners.

Following the thought laid down in the discussion of the treatment of alveolo-dental abscess, it occurred to me years ago that if we desired to cure antral abscess, so called, we must establish an opening sufficiently large to thoroughly evacuate the antral cavity, to clear out the putrid material, to curette the carious walls of the antrum, to remove all diseased structures and all pathological masses, and completely discharge the contents of the antrum, which I have repeatedly found consisted not only of pus, but of *débris* and necrotic substances of a fibrous and osseous nature. The practice sometimes pursued, which, in fact, I regret to say, is very often pursued,—to extract a firm tooth, often a sound tooth, and then perforate the floor of the antrum of Highmore,—is reprehensible in the extreme. In fact, it is an unjustifiable proceeding to sacrifice a useful dental organ, and in the great majority of cases to no purpose, for in almost every case that I have seen, where this line of treatment has been followed in the attempt to cure a case of antral empyema, the opening was entirely inadequate to discharge the contents of the diseased cavity and to admit of satisfactory after-treatment. It has been our practice in the treatment of suppurative diseases of the antrum, especially where they have been of long standing, to raise the soft structures from the anterior antral wall, and then by means of a trephine in the surgical engine make an opening in the antrum large enough to insert the end of the index-finger. Some time ago an eminent member of your profession was present at my clinic and witnessed the operation of trephining the antral wall. He expressed himself as being amazed at the practice of opening the antrum heroically for antral empyema, but his amazement turned to strong commendation when I took out of that diseased antrum, which had been the subject of treatment for over three years, a nodule of inspissated pus and *débris* which was quite as large as a small marble; and when I dislodged, by the lightest touch, a flake of bone larger than a finger-nail, and demonstrated that this flake of bone was not only necrosed but black as the result of long retention, he publicly stated that what he had witnessed had forever changed his views on the treatment of long-continued cases of this kind.

Such facts as these are not as widely known by the dental profession as they should be, but we have established them now in a number of operations, especially within the last three years, and I am to-day prepared to say that it is my belief that the safest and most satisfactory operation for chronic antral abscess is this large anterior opening directly into that cavity, the removal of all pathological masses, the satisfactory irrigation and after-treatment, and, what is invaluable to the patient, no manner of disturbance of the dental arch.

A few suggestions associated with the after-treatment of these cases is of prime importance. In the first place, it is imperatively required to keep the parts antiseptically clean, so as to prevent the accumulation of any discharges or foreign materials. If absolute surgical cleanliness is not observed following this operation, you may have reinfection of the bone and progressive devitalization until a great portion of the maxilla is involved in the process of softening, caries, necrosis, and destruction. The antral cavity must be kept carefully packed and everything done to establish asepsis. In from four to six weeks after the operation the cavity is, as a rule, filled, and, with the extreme care we apply in our after-treatment, I do not recall a single failure to cure antral empyema.

In treating cases of recurrent alveolar disease, as well as cases of chronic antral empyema, we shall continue perforation of the bone into the abscess cavity, thereby insuring the cure of the case and the retention of the dental organs in as perfect a condition as the exigencies of the case will allow.

